# Study of back trunk asymmetry in children from three ethnic groups and correlation with their handedness

**DOI:** 10.1186/1748-7161-7-S1-O74

**Published:** 2012-01-27

**Authors:** TB Grivas, A Kasartzian, C Mazioti, C Mihas, C Aggouris, G Triantafyllopoulos, N Dimitrakos, I Katsoulis

**Affiliations:** 1 Tzanio General Hospital of Piraeus, Piraeus, Greece; 2 Orthopaedic Surgeon, Greece

## Background

Armenian, Albanian and Greek children attending primary and secondary schools in Athens Greece were screened. These three ethnic groups comprise a rather homogeneous genetically population, each of them being culturally a closed social group population. This results in a very interesting group of people from the genetic point of view. The examination and analysis of various anthropometric parameters could reveal and provide useful baseline information for understanding of truncal asymmetry formation, the preliminary condition of spinal deformity.

## Materials and methods

10863 (5372 males and 5491 females), Greek (n=8918), Albanian (n=683) and Armenian (n=265) children and adolescents (5-17 years old) were screened at their school for back truncal asymmetry or scoliosis. Menarche and handedness (laterality) were also documented. The Prujis scoliometer was used to examine the students in standing and sitting forward bending position. Asymmetries were tested for correlation with laterality. These data were fed in SPSS program spreadsheet and statistically analyzed using STATA™ v. 9.0 package.

## Results

The mean menarche was found to be 11,79 years of age.

There is no statistical difference in asymmetry in pre and post menarche Armenian group girls. In sitting forward bending position (sitFBP), comparison of pre-menarche, showed symmetry differences in only the thoracolumbar (ThL) region among Armenian (46.2%), Albanian (88%) and Greek (80,1%) girls (p=0.030), but not post-menarche. In boys aged 5-10 years, in all sitFBP greater symmetry percentages observed in Armenian compared with Albanian and Greek, [in Thoracic (T) p=0.05, Thoracolumbar (ThL)p=0.01, and Lumbar (L) p=0.003].

Comparison of symmetry among girls aged 5-10 years. In all sitFBP greater symmetry percentages in Armenian compared with Albanian and Greek, (in T, ThL and L, p=0.001) were observed. Comparison of symmetry among girls aged 11-17 years reviled difference in right L standing FBP (stdFBP), Armenial 0% asymmetry, Albanian 5.6%, Greek 2.2% respectively (p=0.024), but not for the peered aged boys.

In Armenian group, laterality was correlated with symmetry / asymmetry in L stdFBP, p=0.033. In 11-17 yrs of age Albanian girls, in stdFBP in ThL spine laterality was correlated with asymmetry, p=0.023. In 5-10 yrs old Greek boys correlation between laterality and symmetry - asymmetry in TL spine in sitFBP p=0.015 and stdFBP p=0.049, while in 11-17 yrs old Greek girls, correlation between laterality and symmetry - asymmetry in TL p=0.034 and L p=0.035 spine in stdFBP was found.

## Discussion

The above revealed trunkal asymmetry differences indicate a genetic background. The laterality also seems to dictate in some way the formation of these truncal asymmetries.

